# The Impact of Acne Treatment on Skin Bacterial Microbiota: A Systematic Review

**DOI:** 10.1177/12034754211037994

**Published:** 2021-08-15

**Authors:** Megan Lam, Angie Hu, Patrick Fleming, Charles W. Lynde

**Affiliations:** 1 12362 Michael G. DeGroote School of Medicine, Faculty of Medicine, Hamilton, ON, Canada; 2 2129 Faculty of Science, University of Calgary, AB, Canada; 3 210484 Division of Dermatology, University of Toronto, ON, Canada; 4 Lynde Dermatology (Private Practice), Markham, ON, Canada

**Keywords:** acne, treatment, microbiome, antibiotics, cutibacterium

## Abstract

**Background:**

Microbial strains such as *Cutibacterium acnes* have been examined as contributors to the pathogenesis of acne. Given the prevalence of the disease among adolescents and adults, the overutilization of antimicrobial agents may breed resistance and alter commensal microflora.

**Objectives:**

To characterize the impact of acne treatment on the diversity and relative abundance of the cutaneous microbial community, particularly of the bacterial flora

**Methods:**

An electronic search was conducted of Embase, MEDLINE, and the Cochrane Central Register of Controlled Trials (CENTRAL) on June 5, 2020. Interventional and observational studies examining patients receiving acne treatment with culture-independent, community-level analysis of the cutaneous microbiome were included.

**Results:**

Nine studies with 170 treated acne patients were included. Five studies reported a significant change in alpha diversity following treatment, 3 of which examining systemic antibiotics reported significant increases in diversity. Two of 3 studies examining effects of benzoyl peroxide reported a decrease in diversity. However, trends in diversity were heterogeneous among studies.

**Conclusions:**

While individual variability in microbiome composition, and study-level heterogeneity in study sampling techniques may limit quantitative synthesis, our results support findings that acne treatment, including those not considered to have antimicrobial properties, alters the composition of the cutaneous microbiome.

PROSPERO registration: CRD42020190629

## Introduction

Acne is one of the most common skin diseases, primarily affecting young adults and adolescents, and involving the pilosebaceous unit in a complex interplay of host inflammation, sebum production, hyperkeratinisation of follicles, and colonization of bacteria.^1^ For instance, *Cutibacterium acnes* (*C. acnes*, formerly known as *Propionibacterium acnes*) is a particular target of acne treatment as colonization of *C. acnes* has been shown to promote inflammation in acne patients, among other precipitating factors. However, *C. acnes*, along with other microbial species that induce inflammation related to acne, are also found ubiquitously on healthy skin. Currently, how individual differences in microbial composition affect disease severity remains unclear.^1-3^

There is increasing recognition that commensal microorganisms play an important role in reducing the likelihood of certain skin conditions, and cutaneous microbial dysbiosis has been linked to a weakened external barrier against pathogens.^1,4,5^ The overutilization of antibiotics raises concerns over resistance, and a 2017 study by Barbieri et al. found that 25% of acne vulgaris patients are prescribed oral antibiotics for a duration of longer than 6 months.^6^ Understanding the effect of acne treatments on the cutaneous microbiome can inform clinicians on the unintended results of treatment.

Thus, in this systematic review, we seek to characterize the impact of acne treatment on the cutaneous microbial community, specifically of the bacterial flora, and examine the resultant changes in abundance and diversity of microbial strains.

## Material and Methods

This systematic review was performed in accordance with the Preferred Reporting Items for Systematic Reviews and Meta-Analyses (PRISMA) guidelines. The protocol for this review was prospectively registered on PROSPERO (CRD42020190629).

### Search Strategy

We searched Embase via Ovid, MEDLINE via Ovid, and Cochrane Central Register electronic databases from their respective dates of conception through to June 5, 2020, limiting our search to English records and human studies. Our search strategy is comprised of key terms for acne, acne treatments, and microbiome. Our detailed search strategy can be found in Supplemental Tables S1-S3. Cited studies from included studies and relevant review articles were screened for additional studies not included in the original search.

### Study Selection and Data Abstraction

Interventional and observational studies examining acne patients treated with benzoyl peroxide, topical or systemic antibiotics, and retinoids (isotretinoin or tretinoin) with culture-independent, community-level analysis of the cutaneous microbiome were included. Culture-based methods were excluded due to risk of overrepresentation of bacteria that have a greater tendency to thrive and proliferate in laboratory culture conditions. Studies limiting their investigations to specific taxonomic units were excluded. Studies examining samples from other regions including the nasal cavity or oropharynx were also excluded.

Two investigators (M.L. and A.H.) independently screened titles and abstracts for relevance and assessed full texts for eligibility. The citations of relevant studies and review articles were manually screened for additional citations not identified from electronic searches. Discrepancies between reviewers were discussed until full consensus was reached and senior authors (P.F. and C.W.L.) were consulted if necessary.

We extracted the following data from each study using a standardized form: study characteristics (author, year of publication, country, study design, treatment, methodology), participants demographics (number of participants, age, % female, BMI or obesity, Fitzpatrick skin phototype, race, acne severity), and outcomes (location of samples, taxonomic units reported, bacterial strains with a significant % difference with treatment, alpha diversity and % change).

### Outcomes

The primary outcomes of this review are (i) to characterize the change in community microbiome diversity after acne treatment, (ii) to characterize the changes in relative abundance of bacterial strains following treatment, and (iii) to characterize the changes in relative abundance of *Cutibacterium* following treatment.

The outcome of diversity was measured through alpha diversity, which represents the microbial diversity within an individual sample and can be represented through the Shannon diversity index and the inverse Simpson index, both of which take into account the total number of species in the sample and the proportion of the total sample taken up by each species.

### Quality Assessment of Studies

Risk of bias of studies was assessed using the Newcastle-Ottawa scale for nonrandomized studies and the Cochrane Risk of Bias-2 (ROB-2) tool for randomized controlled trials. The Newcastle-Ottawa scale scores studies out of a total of 9 stars for cohort and case-control studies, while the ROB-2 tool assigns an overall risk of bias of low, moderate, or high based on the risk of bias judgement for each of five domains.

## Results

Our initial literature search yielded a total of 2,672 studies, of which 729 were removed as duplicates (Supplemental Figure S1). A total of 1,943 studies were screened based on titles and abstracts, where 1732 studies were excluded. 211 studies were assessed for eligibility based on full texts. 202 studies were excluded, with most studies excluded based on a lack of community-level analysis (*n* = 95). Other reasons for exclusion include an irrelevant study population (*n* = 18), wrong treatment? (*n* = 17), and using culture-based methods to analyze the microbiome (*n* = 12).

### Study Characteristics

After screening, a total of 9 studies were included with 2 cohort studies, 4 nonrandomized interventional studies, 1 case-control study, and 2 randomized controlled studies. A total of 170 treated acne patients were included with a mean age of 18.4 years and a mean proportion female participants of 75%. Four studies included a healthy control comparison group, and 31 healthy control patients were included. One study included an untreated acne group of 4 patients. Two studies included only pediatric subjects, ranging from 7 to 12 years of age. Three studies included only females in their treatment group.

Five studies reported Fitzpatrick skin phototype for their participants and 5 studies reported participant race (Supplemental Table S4).

Eight out of 9 studies collected samples using cotton swabs and all studies included skin samples from cheeks. All studies used 16S rRNA sequencing, targeting the V1-V2,^7^ V1-V3,^8,9^ V3-V4,^4,10-12^ and V4 regions.^13^

A detailed summary of study and participant characteristics can be found in Supplemental Table S5.

### Quality Assessment of Studies

For nonrandomized studies assessed using the Newcastle-Ottawa scale, all studies were found to have a risk of bias rating of 5 stars or above (5 stars,^4,8,11,12^ 6 stars,^14^ 7 stars^7,10^). For randomized controlled studies assessed using the ROB-2 tool, 1 moderate^9^ and one low^13^ risk of bias judgement was assigned. A summary of scoring distribution for risk of bias of included studies can be found in Supplemental Table S6.

### Primary Outcomes

In total, 8 of the 9 included studies reported changes in alpha diversity following treatment, where 7 studies used the Shannon index and 2 studies used both the Shannon and Inverse Simpson indices.

Taxonomic strains that were reported to show statistically significant changes in abundance with treatment can be found in Supplemental Table S7. Given the heterogeneity between studies, meta-analyses of the data were unable to be performed and qualitative rather than quantitative synthesis was performed. A summarized overview of study outcomes can be found in Supplemental Table S8.

#### Antibiotics

Four studies examined the microbiome before and after treatment with systemic or oral antibiotics, including minocycline,^4,11^ doxycycline,^10^ and lymecycline.^8^ Two of these studies included healthy control groups,^8,11^ and one of these studies compared the use of oral lymecycline with isotretinoin.^8^

All 4 studies examining changes to the skin microbiome with oral antibiotic use reported alpha diversity measurements. Overall, 3 of the studies found an increase in alpha diversity, and 1 reported a decrease.

Two studies found a statistically significant increase in alpha diversity of acne patients’ microbiota following treatment in both the Shannon and Inverse Simpson indices.^8,10^ Park et al. reported a significant increase in alpha diversity in the after-treatment group, compared to before treatment, following 6 weeks of oral doxycycline (Shannon index, 1.27-fold increase, *P* = .03, 95% CI 0.1-1.4; Inverse Simpson, 1.11-fold increase, *P* = .03, 95% CI 0.005-0.014).^10^ Kelhala et al. found significant increases in alpha diversity after treatment in back (*P* ≤ .05) and cheek samples (*P* ≤ .01) following 6 weeks of treatment with either lymecycline or isotretinoin, although the specific treatment was not specified in the study for this analysis. However, the study also found a significant decrease in diversity in armpit samples after treatment (*P* ≤ .01).^8^ Another study by Thompson et al. reported increased alpha diversity in comparison to baseline levels (*P* = .153) and acne-free controls (*P* = .264) using the Shannon index, although the changes were not statistically significant.^11^ In contrast, Chien et al. reported a decrease in diversity following antibiotic treatment and while the overall change was not significant, analyses of individual patients revealed statistically significant decreases in alpha diversity for 2 of its 4 participants.^4^

Three of the 4 studies reported a significant decrease in abundance of the *Cutibacterium* genus.^4,8,10^ Two of the studies reported a significant decrease in *C. acnes* abundance from baseline to following treatment,^4,10^ and the study by Thompson et al. reported a significant decrease in *C. acnes* in the after-treatment group compared to healthy controls.^11^

The study by Dreno et al. examining topical 4% erythromycin use did not report changes in alpha diversity following treatment, but found a significant decrease in *Cutibacterium* in comedones following treatment.^13^

#### Retinoids

A total of 3 studies investigated the use of retinoids, where 2 studies examined isotretinoin treatment,^7,8^ while 1 study used tretinoin.^9^

The 2 studies examining treatment with isotretinoin reported increases in alpha diversity, while the study by Coughlin et al. which used topical tretinoin found a decrease in diversity. Coughlin et al. reported a significant decrease in alpha diversity to level similar to control participants, based on number of observed species and phylogenetic diversity, following 7 to 10 weeks of treatment with tretinoin or BP.^9^

Both studies examining treatment with isotretinoin reported decreases in abundance of *Cutibacterium*. Kelhala et al. reported a significant decrease in *Cutibacterium* levels,^8^ and similarly, McCoy et al. found relative abundance of *Cutibacterium* to be significant less at all time points following treatment compared to untreated acne and control groups.^7^

The study by Coughlin et al. which examined topical tretinoin did not report overall changes in relative abundance, or statistical significance, following treatment.^9^

#### Benzoyl Peroxide

Three studies examined treatment with benzoyl peroxide products and 2 of the studies reported a decrease in phylogenetic (alpha) diversity following treatment.^9,12,14^ Coughlin et al. found a significant decrease in diversity based on the number of observed species and phylogenetic diversity. The decrease in diversity reported by Ahluwalia et al. was not significant (*P* = .368). The study by Karoglan et al. was the only study examining benzoyl peroxide to find an increase in diversity following treatment from 2.3 to 2.6 using the Shannon diversity index, but did not report statistical significance.

Coughlin et al. and Karoglan et al. reported a decrease in relative abundance of *Cutibacterium* following treatment with BP. Ahluwalia et al. did not report overall changes in relative abundance, or statistical significance, following treatment.

### Microbiome Diversity of Acne Skin Compared to Healthy Controls

Alpha diversity of acne skin samples compared to healthy control groups was reported in 4 studies.^7-11,11^ Three of the 4 studies reported no significant differences between untreated acne patients and controls with respect to the Shannon index of alpha diversity,^7,8,11^ with the fourth study reporting a higher alpha diversity in untreated acne patients compared to controls.^9^ However, in general, notable differences were observed in the relative abundance of several microbial taxa, including *Cutibacterium*.^7,8^

## Discussion

This systematic review highlights the impact of acne treatment in altering the host microflora, including therapies which are not conventionally associated with antimicrobial properties such as topical retinoids. Alterations in the diversity of community microbes and the relationship between resident microorganisms and the host response are important considerations in the treatment of acne, particularly given the link between the disease and dysbiosis. This mutualistic relationship and equilibrium of the microbiome have been documented to contribute to the health of the host skin, where the loss of diversity has been associated with chronic inflammatory skin conditions including atopic dermatitis, psoriasis and acne.^5,13^

The composition of one’s skin flora varies with past treatment exposure, age, and differs depending on body site,^15-17^ as does one’s responsiveness to antibiotic treatment.^9^ It is possible that these individual variances contributed to the heterogeneity of the results reported in our included studies.

The increase in microbial diversity following treatment could have been attributed to a decrease in relative abundance of *C. acnes*, allowing other microbial strains to proliferate.^8^ Most studies included in this review reported a decrease in *C. acnes*, and 1 study in particular found that a negative correlation between *C. acnes* levels and pseudomonas species levels,^4^ suggesting that the strains, along with others, may be competing for the same niche environment. The role of commensal *C. acnes* in inhibiting the invasion of pathogenic strains such as staphylococcus aureus also suggests a mechanism of niche competition.^5,13,18^ The duration of the follow-up period would have influenced the degree of change in relative abundance of species, and thereby the overall diversity of the microbiome. While most included studies had a follow-up period of 4 to 6 weeks, the studies with the longest (5, 7 months),^3^ and the shortest (1 week)^4^ follow-up periods both noted an increase in diversity, suggesting that multiple factors, in addition to the antimicrobial properties of treatment, are involved; however, additional data is needed to draw firm conclusions.

While benzoyl peroxide has antibacterial and comedolytic properties, the effects of topical and systemic retinoic acids on microbial activity is not as well established. A study by Oprica et al. reported that isotretinoin demonstrated superior antimicrobial efficacy in relation to *C. acnes* abundance, compared to tetracycline.^19^ In this review, the studies examining isotretinoin treatment found a significant difference in bacterial diversity following treatment. Additionally, Coughlin et al. states that the decrease in bacterial diversity found with treatment with topical tretinoin suggests that topical retinoids may indeed influence the cutaneous microenvironment, thus altering the composition of resident microorganisms.^9^

### Limitations

Our study had several limitations. First, the major limiting factor contributing to heterogeneity between studies is variations in methodology between studies, particularly where different regions of the 16S gene were sequenced in different studies. Additionally, 8 out of 9 studies included used skin swabs, which may have failed to sample the bacterial community in the pilosebaceous follicles.^4,10^ However, a 2018 study by Hall et al. that surface and follicular sampling methods demonstrated no difference in *C. acnes* associated factors.^20^ Second, this review examined on intra-sample diversity (alpha diversity) to characterize the effects of acne treatment, but further exploration on individual- and treatment-level, inter-sample diversity (beta diversity) may yield additional insight into how individual variability may impact treatment influence on microbiota. The studies examined in this review included acne patients of all ages, skin types, and geographic regions, variability between participants made firm conclusions difficult. Despite the high prevalence of acne in adolescent and adult populations world-wide, the literature examining the interdependence between bacterial flora and acne pathogenesis is limited, particularly with studies examining the change in microbiome following topical retinoid usage.

### Conclusion

Acne treatment plays a complex role in influencing the composition of the cutaneous microbiome, including systemic antibiotics and treatments not conventionally associated with antibacterial properties. , The heterogeneity of included studies made forming meaningful conclusions difficult, and highlights the need for future studies using high-quality methodologies, clear metadata fields, and extended sampling to better characterize long-term impacts on the cutaneous microbiome following acne treatments.

## Supplemental Material

Online supplementary file 1 - Supplemental material for The Impact of Acne Treatment on Skin Bacterial Microbiota: A Systematic Review Click here for additional data file. Supplemental material, Online supplementary file 1, for The Impact of Acne Treatment on Skin Bacterial Microbiota: A Systematic Review by Megan Lam, Angie Hu, Patrick Fleming and Charles W. Lynde in Journal of Cutaneous Medicine and Surgery

## References

[1] O’Neill AM. Gallo RL . Host-Microbiome interactions and recent progress into understanding the biology of acne vulgaris. Microbiome. 2018;6(1):1-16. 30285861 10.1186/s40168-018-0558-5 PMC6169095

[bibr2-12034754211037994] Jończyk-Matysiak E. Weber-Dąbrowska B. Żaczek M et al. Prospects of phage application in the treatment of acne caused by propionibacterium acnes. Front Microbiol. 2017;8 10.3389/fmicb.2017.00164 PMC5296327 28228751

[3] Shaheen B. Gonzalez M . A microbial aetiology of acne: what is the evidence? Br J Dermatol. 2011;165(3):474-485. 10.1111/j.1365-2133.2011.10375.x 21495996

[4] Chien AL. Tsai J. Leung S et al. Association of systemic antibiotic treatment of acne with skin microbiota characteristics. JAMA Dermatol. 2019;155(4):425-434. 10.1001/jamadermatol.2018.5221 30758497 PMC6459106

[5] Grice EA. Segre JA . The skin microbiome. Nat Rev Microbiol. 2011;9(4):244-253. 10.1038/nrmicro2537 21407241 PMC3535073

[6] Barbieri JS. James WD. Margolis DJ . Trends in prescribing behavior of systemic agents used in the treatment of acne among dermatologists and nondermatologists: a retrospective analysis, 2004-2013. J Am Acad Dermatol. 2017;77(3):456-463. 10.1016/j.jaad.2017.04.016 28676330

[7] McCoy WH. Otchere E. Rosa BA. Martin J. Mann CM. Mitreva M . Skin ecology during sebaceous drought - How skin microbes respond to isotretinoin. J Invest Dermatol. 2019;139(3):732-735. 10.1016/j.jid.2018.09.023 30579608 PMC6905187

[bibr8-12034754211037994] Kelhälä H-L. Aho VTE. Fyhrquist N et al. Isotretinoin and lymecycline treatments modify the skin microbiota in acne. Exp Dermatol. 2018;27(1):30-36. 10.1111/exd.13397 28636791

[9] Coughlin CC. Swink SM. Horwinski J et al. The preadolescent acne microbiome: a prospective, randomized, pilot study investigating characterization and effects of acne therapy. Pediatr Dermatol. 2017;34(6):661-664. 10.1111/pde.13261 29024079

[10] Park S-Y. Kim HS. Lee SH. Kim S . Characterization and analysis of the skin microbiota in acne: impact of systemic antibiotics. J Clin Med. 2020;9(1):168. 10.3390/jcm9010168 31936262 PMC7019264

[bibr11-12034754211037994] Thompson KG. Rainer BM. Antonescu C et al. Minocycline and its impact on microbial dysbiosis in the skin and gastrointestinal tract of acne patients. Ann Dermatol. 2020;32(1):21-30. 10.5021/ad.2020.32.1.21 33911705 PMC7992645

[12] Karoglan A. Paetzold B. Lima J et al. Safety and efficacy of topically applied selected Cutibacterium acnes strains over five weeks in patients with acne vulgaris: an open-label, pilot study. Acta Derm Venereol. 2019;99(13):1253-1257. 10.2340/00015555-3323 31573666

[13] Dreno B. Martin R. Moyal D. Henley JB. Khammari A. Seité S . Skin microbiome and *acne vulgaris*: *Staphylococcus*, a new actor in acne. Exp Dermatol. 2017;26(9):798-803. 10.1111/exd.13296 28094874

[14] Ahluwalia J. Borok J. Haddock ES et al. The microbiome in preadolescent acne: assessment and prospective analysis of the influence of benzoyl peroxide. Pediatr Dermatol. 2019;36(2):200-206. 10.1111/pde.13741 30656737

[15] Grice EA. Kong HH. Conlan S et al. Topographical and temporal diversity of the human skin microbiome. Science. 2009;324(5931):1190-1192. 10.1126/science.1171700 19478181 PMC2805064

[bibr16-12034754211037994] Johnson CL. Versalovic J . The human microbiome and its potential importance to pediatrics. Pediatrics. 2012;129(5):950-960. 10.1542/peds.2011-2736 22473366 PMC3340594

[17] Oh J. Conlan S. Polley EC. Segre JA. Kong HH . Shifts in human skin and nares microbiota of healthy children and adults. Genome Med. 2012;4(10):77. 10.1186/gm378 23050952 PMC3580446

[18] Ladizinski B. McLean R. Lee KC. Elpern DJ. Eron L . The human skin microbiome. Int J Dermatol. 2014;53(9):1177-1179. 10.1111/ijd.12609 25070027

[19] Oprica C. Emtestam L. Hagstromer L. Nord CE . Clinical and microbiological comparisons of isotretinoin vs. tetracycline in acne vulgaris. Acta Derm Venereol. 2007;87(3):246-254. 17533492 10.2340/00015555-0211

[20] Hall JB. Cong Z. Imamura-Kawasawa Y et al. Isolation and identification of the follicular microbiome: implications for Acne Research. J Invest Dermatol. 2018;138(9):2033-2040. 10.1016/j.jid.2018.02.038 29548797

