# Supplementary material for: The Impact of Acne Treatment on Skin Bacterial Microbiota: A Systematic Review
Source: J Cutan Med Surg. 2021 Aug 15;26(1):93–7. doi: 10.1177/12034754211037994 (PMC8750125; doi:10.1177/12034754211037994)
Supplement: Online supplementary file 1 - Supplemental material for The Impact of Acne Treatment on Skin Bacterial Microbiota: A Systematic Review [file sj-docx-1-cms-10.1177_12034754211037994.docx]

SUPPLEMENTAL MATERIAL

**Table S1**: MEDLINE search strategy

| # | searches | results |
| --- | --- | --- |
| 1 | exp Acne Vulgaris/ | 11613 |
| 2 | acne.mp. | 18393 |
| 3 | 1 or 2 | 18393 |
| 4 | exp Tretinoin/ | 22110 |
| 5 | Isotretinoin/ | 3545 |
| 6 | isotretino*.mp. | 4573 |
| 7 | exp Retinoids/ | 55394 |
| 8 | exp Salicylic Acid/ | 8441 |
| 9 | exp Anti-Bacterial Agents/ | 723738 |
| 10 | antibio*.mp. | 387559 |
| 11 | antibacteri*.mp. | 76295 |
| 12 | treatment*.mp. | 5003618 |
| 13 | retino*.mp. | 149253 |
| 14 | Benzoyl peroxide/ | 1096 |
| 15 | benzoyl peroxide.mp. | 1823 |
| 16 | 4 or 5 or 6 or 7 or 8 or 9 or 10 or 11 or 12 or 13 or 14 or 15 | 5779178 |
| 17 | exp Microbiota/ | 33592 |
| 18 | Bacteria/ | 147173 |
| 19 | microflora.mp. | 15771 |
| 20 | microb*.mp. | 1180026 |
| 21 | Propionibacterium acnes/ | 3778 |
| 22 | propionibacter*.mp. | 7668 |
| 23 | cutibacterium acnes.mp. | 236 |
| 24 | cutibacter*.mp. | 295 |
| 25 | bacillus acnes.mp. | 1 |
| 26 | 17 or 18 or 19 or 20 or 21 or 22 or 23 or 24 or 25 | 1238477 |
| 27 | 3 and 16 and 26 | 1317 |
| 28 | limit 27 to (english language and humans) | **942** |

Note: / - MeSH search, * - truncation, exp – explode

**Table S2**: Embase search strategy

| # | searches | results |
| --- | --- | --- |
| 1 | exp acne/ | 33169 |
| 2 | acne.mp. | 36475 |
| 3 | 1 or 2 | 37424 |
| 4 | exp benzoyl peroxide/ | 4274 |
| 5 | exp anti acne agent/ | 72601 |
| 6 | retinoid/ | 14416 |
| 7 | retino*.mp. | 256854 |
| 8 | salicylic acid/ | 23182 |
| 9 | treatment*.mp. | 7064635 |
| 10 | retinoic acid/ | 41912 |
| 11 | tretinoin.mp. | 2215 |
| 12 | isotretinoin/ | 12586 |
| 13 | isotretino*.mp. | 12917 |
| 14 | exp antibiotic agent/ | 1446997 |
| 15 | antibio*.mp. | 758549 |
| 16 | antibacteri*.mp. | 131427 |
| 17 | 4 or 5 or 6 or 7 or 8 or 9 or 10 or 11 or 12 or 13 or 14 or 15 or 16 | 8264235 |
| 18 | microbiome/ | 16540 |
| 19 | microbio* | 529597 |
| 20 | microflora/ | 20003 |
| 21 | bacterial microbiome/ | 803 |
| 22 | Propionibacterium acnes/ | 8202 |
| 23 | propionibacter*.mp. | 12497 |
| 24 | cutibacterium acnes.mp. | 271 |
| 25 | cutibacter*.mp. | 342 |
| 26 | bacillus acnes.mp. | 1 |
| 27 | 18 or 19 or 20 or 21 or 22 or 23 or 24 or 25 or 26 | 544947 |
| 28 | 3 and 17 and 27 | 2256 |
| 29 | limit 28 to (human and english language) | **1539** |

Note: / - MeSH search, * - truncation, exp – explode

**Table S3**: Cochrane Central Register search strategy

| # | searches | results |
| --- | --- | --- |
| 1 | MeSH descriptor: [Acne Vulgaris] explode all trees | 1328 |
| 2 | acne | 4394 |
| 3 | #1 or #2 | 4394 |
| 4 | MeSH descriptor: [Benzoyl Peroxide] explode all trees | 308 |
| 5 | MeSH descriptor: [Retinoids] explode all trees | 2468 |
| 6 | retino* | 10400 |
| 7 | MeSH descriptor: [Tretinoin] explode all trees | 722 |
| 8 | isotretino* | 612 |
| 9 | treatment* | 756025 |
| 10 | MeSH descriptor: [Anti-Bacterial Agents] explode all trees | 11903 |
| 11 | salicylic acid | 1039 |
| 12 | antibiotic | 22428 |
| 13 | antibacteri* | 13360 |
| 14 | #4 or #5 or #6 or #7 or #8 or #9 or #10 or #11 or #12 or #13 | 770637 |
| 15 | MeSH descriptor: [Microbiota] explode all trees | 619 |
| 16 | microflora | 1867 |
| 17 | bacterial microbiome | 725 |
| 18 | Propionibacterium acnes | 289 |
| 19 | microbio* | 24994 |
| 20 | propionibacteri* | 436 |
| 21 | cutibacteri*.mp. | 19 |
| 22 | #15 or #16 or #17 or #18 or #19 or #20 or #21 | 25981 |
| 23 | #3 and #14 and #22 | 215 |
| 24 | #23 (limit to trials) | **191** |

**Figure S1:** Study selection methodology

**
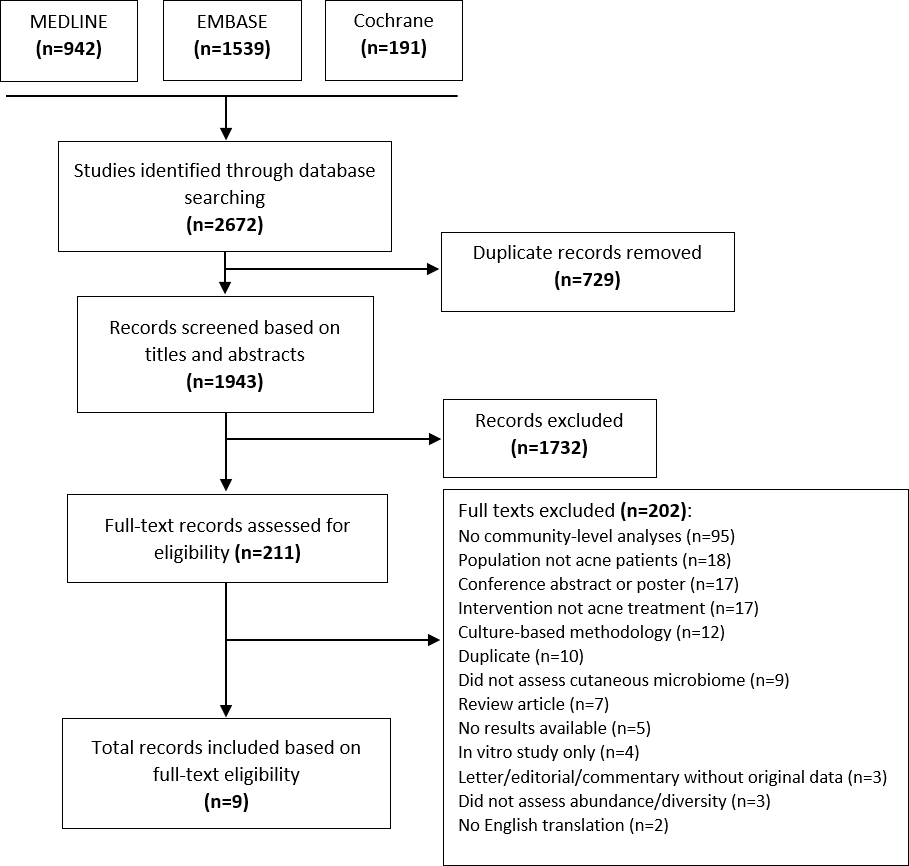
**

**Table S4:** Overview of participant characteristics

| **Study** | ***N (treatment)*** | **Mean age (SD)** | **Age range** | **% female** | **Fitzpatrick skin phototype** | **Race** |
| --- | --- | --- | --- | --- | --- | --- |
| Park et al., 2020 | 20 acne treated (doxycycline) | 19.6 (7.7) | 11-44 | 50% | median 4 (range 3-5) | Asian (n=20) |
| Thompson, 2020 | 8 acne treated (minocycline) | - | 20-32 | - | - | African American (n=3), Caucasian (n=3), Asian (n=2) |
|  | 8 controls | - | 23-34 | - | - | - |
| Chien, 2019 | 4 acne treated (minocycline) | 28.5 (4.7) | 25-35 | 100% | II (n=2), III (n=1), V (n=1) | White (n=2), African American (n=1), Asian (n=1) |
| Kelhala, 2017 | 9 acne treated (lymecycline) | 19.0 (3.0) | 15-23 | 67% | - | All Caucasian |
|  | 13 acne treated (isotretinoin) | 23.9 (8.3) | 15-41 | 46% | - | All Caucasian |
|  | 11 controls | 24.2 (3.1) | 19-30 | 64% | - | All Caucasian |
| Dreno, 2017 | 26 acne treated (erythromycin) | 24 (6.5) | - | 58% | II (n=11), III (n=13), IV (n=1), VI (n=1) | - |
| McCoy, 2019 | 17 acne treated (isotretinoin) | 21.2 | 13-41 | 94% | 2.8 | - |
|  | 4 acne untreated | 25.3 | 21-32 | 75% | 2.8 | - |
|  | 4 controls | 24.0 | 21-28 | 100% | 2.5 | - |
| Coughlin, 2017 | 5 acne treated (benzoyl peroxide) | 9 | 7-10 | 60% | - | - |
|  | 3 acne treated (tretinoin) | 9 | 7-10 | 100% | - | - |
|  | 8 controls | 9 | 7-10 | - | - | - |
| Ahluwalia, 2019 | 51 acne treated (benzoyl peroxide) | 10 | 7-12 | 100% | I (n=1), II (n=10), III (n=11), IV (n=25), V (n=4) | Non-hispanic White (n=26); Hispanic/Latino (n=23); Asian/Pacific Islander (n=1); Black (n=1) |
| Karoglan, 2019 | 14 acne treated (benzoyl peroxide) | 19.8 | 18-23 | - | - | - |

Abbreviations: SD, Standard Deviation

**Table S5:** Overview of study characteristics

| **Study** | **Participant source; country** | **Study design** | **Treatment** | **Sampling** | **Location of samples** | **Methodology** | **Length of follow-up** |
| --- | --- | --- | --- | --- | --- | --- | --- |
| Park, 2020 | Patients enrolled in Aug 2017 to June 2018 at the Department of Dermatology, Incheon St. Mary’s Hospital; Korea | Non-randomized interventional | Doxycycline 100 mg twice daily for 6 weeks | 4 cm^2^ areas with cotton swabs; rubbed 20 times | Skin of bilateral cheeks | V3-V4 hypervariable region of the 16s rRNA gene was PCR-amplified; sequenced on Illumina HiSeq platform | 6 weeks |
| Thompson, 2020 | Moderate-to-severe acne vulgaris patients matched to acne-free controls; USA | Case-control | Oral minocycline (100 mg twice daily) for 4 weeks | Cotton swab, rubbed for 30 seconds | Skin of bilateral cheeks, nose, and chin | V3-V4 hypervariable region of the 16s rRNA gene was PCR-amplified; sequenced on Illumina MiSeq platform | 4 weeks |
| Chien, 2019 | Women with recent diagnosis of acne enrolled from Feb 11 to Sept 23, 2014 at Johns Hopkins Department of Dermatology, Baltimore, Maryland; USA | Cohort | Minocycline, 100 mg, taken orally twice daily for 4 weeks | Skin swabs | Skin of bilateral forehead, cheek, chin | V3-V4 hypervariable region of the 16S rRNA gene was PCR-amplified; sequenced on Illumina MiSeq platform | 4 weeks |
| Kelhälä, 2018 | Acne patients recruited from the dermatology outpatient clinic at the Oulu University Hospital; Finland | Non-randomized interventional | Isotretinoin: 0.4-0.6 mg/kg/d  Lymecycline: 300 mg twice daily | 4 cm^2^ areas with cotton swabs; rubbed 20 times | Skin of cheek, back, and armpit | V1-V3 region of the 16s rRNA gene PCR-amplified with universal bacterial primers; sequenced on Illumina MiSeq platform | 6 weeks |
| Dreno, 2016 | Study conducted between March and June 2014; France | Randomized Controlled Trial | Topical 4% erythromycin | 9 mm^2^ areas with cotton swabs; rubbed for 20 seconds | Skin of cheeks, forehead, temple, chin | V4 region of 16s rRNA gene was PCR-amplified using 515F and 806R primers; sequenced on Illumina MiSeq platform | 4 weeks |
| McCoy, 2019 | Patients recruited from Washington University School of Medicine dermatology clinics from 2015-2017; USA | Cohort | Isotretinoin 0.5-1 mg/kg/day over 5-7 months | Skin swab | For acne patients: skin of forehead, cheek, chin, jawline; for controls: bilateral malar cheeks | V1-V2 region of 16s rRNA gene was PCR-amplified with 16S primers; sequenced on Illumina MiSeq instrument | 5-7 months |
| Coughlin, 2017 | Children with acne that would be appropriately treated with topical regimen; study conducted March to August 2015; USA | Randomized Controlled Trial | Benzoyl Peroxide 5% gel/cream, or tretinoin 0.025% | Pore strips moistened with DNA-free water | Midline forehead, dorsum of the nose, medial left cheek, chin, left retroauricular crease | V1-V3 region of the 16s rRNA gene PCR-amplified using 27F and 534R primers; sequenced on Illumina MiSeq platform | 7-10 weeks (mean 8.6 weeks) |
| Ahluwalia, 2019 | Girls with acne vulgaris from the outpatient dermatology clinic from April 2016 to June 2017; USA | Non-randomized interventional | Benzoyl Peroxide wash nightly | Skin swabs | Skin of forehead, cheeks, nose, chin, retroauricular crease | Proderm IQ microbiome profiling panel that analyzed V1, V2, V4, V6, V7, V8, and V9 regions of 16S rRNA gene; amplified by PCR; sequenced on Illumina MiSeq platform | 4-8 weeks |
| Karoglan, 2019 | Patients with mild-to-moderate acne vulgaris; Germany | Non-randomized interventional | 5% benzoyl peroxide gel | Swab rubbed over skin for 30 seconds | - | SLST region and 16S rRNA V3-V4 were amplified and sequenced on MiSeq v3 | 1 week |

**Table S6:** Quality assessment of included nonrandomized and randomized studies

|  | ***Risk of Bias for nonrandomized studies*** | | | | | |
| --- | --- | --- | --- | --- | --- | --- |
| ***Study*** | *Selection* | | *Comparability* | | *Outcome* | |
| Ahluwalia, 2019 | 2 | | 2 | | 2 | |
| Chien, 2019 | 1 | | 2 | | 2 | |
| Karoglan, 2019 | 1 | | 2 | | 2 | |
| Kelhala, 2017 | 2 | | 1 | | 2 | |
| McCoy, 2017 | 4 | | 2 | | 1 | |
| Park, 2020 | 3 | | 2 | | 2 | |
| Thompson, 2020 | 1 | | 2 | | 2 | |
|  | ***Risk of Bias for randomized studies*** | | | | | |
| ***Study*** | *Domain 1* | *Domain 2* | *Domain 3* | *Domain 4* | *Domain 5* | *Overall* |
| Coughlin, 2017 | Low | Moderate | NI | Low | Moderate | Moderate |
| Dreno, 2017 | Low | Low | Low | Low | Low | Low |

**Table S7**: Significant change in relative abundance of bacterial strains after treatment

| **Study** | **Taxonomic Unit** | **Strain** | **% change, p-value, CI** |
| --- | --- | --- | --- |
| *Park, 2020* | Genus | Cutibacterium | 1.81-fold decrease, p=0.02, 2.3%-2.1% |
|  |  | Snograssella | 3.85-fold decrease, p=0.006, 0.2%-24% |
|  | Species | Cutibacterium acnes | 1.96-fold decrease, p=0.02, 3%-22% |
|  |  | Cutibacterium granulosum | 4.46-fold increase, p=0.02, 0.04%-0.9% |
|  |  | Snodgrassella alvi | 3.85-fold decrease, p=0.006; 0.2%-24% |
| *Thompson, 2020* | Species | Blautia obeum | Increase, p=0.001 |
|  |  | Eubacterium dolichum | Increase, p=0.001 |
|  |  | Klebsiella oxytoca | Increase, p=0.001 |
|  |  | Comamonas terrigena | Increase, p=0.013 |
|  |  | Roseburia faecis | Increase, p=0.015 |
|  |  | Bifidobacterium longum | Increase, p=0.028 |
|  |  | Leuconostoc mesenteroides | Increase, p=0.029 |
|  |  | Dorea formicigenerans | Increase, p=0.041 |
|  |  | Bacillus flexus | Decrease, p=0.001 |
|  |  | Paracoccus marcusii | Decrease, p=0.001 |
|  |  | Actinobacillus parahaemolyticus | Decrease, p=0.001 |
|  |  | Acinetobacter rhizophaerae | Decrease, p=0.001 |
|  |  | Staphylococcus epidermidis | Decrease, p=0.009 |
|  |  | Prevotella nigrescens | Decrease, p=0.028 |
| *Chien, 2019* | Genus | Cutibacterium | 10.3% decrease, p=0.04, -19.9% to -0.7% |
|  |  | Corynebacterium | 2.4% decrease, p=0.08, -1.3% to 0.1% |
|  |  | Prevotella | 0.7% decrease, p=0.008, -1.3% to 0.1% |
|  |  | Porphyromonas | 0.5% decrease, p=0.01, -0.9% to -0.1% |
|  |  | Finegoldia | 0.1% increase, p=0.03, 0.01% to 0.2% |
|  |  | Pseudomonas | 2.2% increase, p<0.001, 0.9% to 3.4% |
|  |  | Erwinia | 1.6% increase, p<0.001, 0.6% to 2.6% |
|  |  | Actinobacillus | 0.6% increase, p=0.04, 0.04% to 1.2% |
|  |  | Micrococcus | 3.8% increase, p=0.03, 0.7% to 6.9% |
|  | Species | Cutibacterium acnes | Decrease, p<0.05 |
| *Kelhala, 2017 (isotretinoin)* | Genus | Cutibacterium* | -2.77, p=0.00000315 |
|  | Family | Propionibacteriaceae | -2.55, p=0.000000353 |
|  |  | Streptococcaceae | 1.21, p=0.0278 |
|  |  | Pasteurellaceae | 2.40, p=0.000826 |
|  |  | Fusobacteriaceae | 2.06, p=0.0278 |
| *(lymecycline - cheek)* | Genus | Cutibacterium* | -2.58, p=0.00506 |
|  | Family | Propionibacteriaceae | -2.22, p=0.0000580 |
| *(lymecycline – back)* | Family | Propionibacteriaceae | -2.58, p=0.000942 |
| *Dreno, 2017* | Phylum | Actinobacteria (comedones) | -2.96% decrease, p<0.05 |
|  | Genus | Corynebacterium (comedones) | -2.86% decrease, p<0.05 |
|  |  | Cutibacterium* (comedones) | -0.44% decrease, p<0.1 |
|  |  | Staphylococcus (papulo-pustular) | -10.41% decrease, p<0.1 |
| *McCoy, 2019* | Genus (compared to controls) | Cutibacterium* | Lower in post-treatment, p<0.05 |
|  |  | Pedobacter | Higher in post-treatment, p<0.05 |
|  |  | Gemelli | Higher in post-treatment, p<0.05 |
|  |  | Prevotella | Higher in post-treatment, p<0.05 |
|  |  | Sediminibacterium | Higher in post-treatment, p<0.05 |
|  |  | Arthrobacter | Higher in post-treatment, p<0.05 |
|  |  | Aerococcus | Higher in post-treatment, p<0.05 |
|  |  | Streptococcus | Higher in post-treatment, p<0.05 |
|  |  | Lachnospiracea | Higher in post-treatment, p<0.05 |
|  |  | Fusobacterium | Higher in post-treatment, p<0.05 |
|  |  | Tepidimonas | Higher in post-treatment, p<0.05 |
|  |  | Peptostreptococcus | Higher in post-treatment, p<0.05 |
|  |  | Porphyromonas | Higher in post-treatment, p<0.05 |
|  |  | Eubacterium | Higher in post-treatment, p<0.05 |
|  | Genus (compared to acne untreated) | Cutibacterium* | Lower in post-treatment, p<0.05 |
|  |  | Pedobacter | Higher in post-treatment, p<0.05 |
|  |  | Gemelli | Higher in post-treatment, p<0.05 |
|  |  | Prevotella | Higher in post-treatment, p<0.05 |
|  |  | Sediminibacterium | Higher in post-treatment, p<0.05 |
|  |  | Acidovorax | Higher in post-treatment, p<0.05 |
|  |  | Cryobacterium | Higher in post-treatment, p<0.05 |
|  |  | Arcicella | Higher in post-treatment, p<0.05 |
|  |  | Solobacterium | Higher in post-treatment, p<0.05 |
|  |  | Flavobacterium | Higher in post-treatment, p<0.05 |
|  |  | Paludibacter | Higher in post-treatment, p<0.05 |
|  |  | Chryseobacterium | Higher in post-treatment, p<0.05 |
|  |  | Polynucleobacter | Higher in post-treatment, p<0.05 |
|  |  | Citrobacter | Higher in post-treatment, p<0.05 |
|  |  | Rothia | Higher in post-treatment, p<0.05 |
|  |  | Actinomyces | Higher in post-treatment, p<0.05 |
|  |  | Sphingobacterium | Higher in post-treatment, p<0.05 |
|  |  | Arthrobacter | Higher in post-treatment, p<0.05 |
|  |  | Aerococcus | Higher in post-treatment, p<0.05 |
|  |  | Streptococcus | Higher in post-treatment, p<0.05 |
|  |  | Lachnospiracea | Higher in post-treatment, p<0.05 |
|  |  | Fusobacterium | Higher in post-treatment, p<0.05 |
|  |  | Tepidimonas | Higher in post-treatment, p<0.05 |
|  |  | Delfia | Higher in post-treatment, p<0.05 |
|  |  | Corynebacterium | Higher in post-treatment, p<0.05 |
|  |  | Mycobacterium | Higher in post-treatment, p<0.05 |
|  |  | Nocardioides | Higher in post-treatment, p<0.05 |
|  |  | Enterococcus | Higher in post-treatment, p<0.05 |
|  |  | Clostridium | Higher in post-treatment, p<0.05 |
|  |  | Diaphorobacter | Higher in post-treatment, p<0.05 |
|  |  | Klebsiella | Higher in post-treatment, p<0.05 |
|  |  | Pseudomonas | Higher in post-treatment, p<0.05 |
|  |  | Haliscomenobacter | Higher in post-treatment, p<0.05 |
|  |  | Gordonia | Higher in post-treatment, p<0.05 |
|  |  | Moryella | Higher in post-treatment, p<0.05 |
|  |  | Pelomonas | Higher in post-treatment, p<0.05 |
|  |  | Methylotenera | Higher in post-treatment, p<0.05 |
|  |  | Legionella | Higher in post-treatment, p<0.05 |
|  |  | Acinetobacter | Higher in post-treatment, p<0.05 |
| *Coughlin, 2017* | - | - | - |
| *Ahluwalia, 2019* | - | - | - |
| *Karoglan, 2019* | - | - | - |

*Cutibacterium reported as Propionibacterium

**Table S8:** Acne severity and microbial diversity following treatment

| **Study** | **No. of participants (treatment)** | ***Acne Severity*** | | | ***Alpha diversity*** | | | ***Cutibacterium*** | |
| --- | --- | --- | --- | --- | --- | --- | --- | --- | --- |
|  |  | *Scale* | *Baseline* | *Post-treatment* | *Index* | *Change after treatment* | *Signif-*  *icance* | *Relative abundance* | *p-value* |
| Park, 2020 | 20 (doxycycline) | IGA (median) | 3 | 2 | Shannon | Increased 1.27-fold compared to baseline | * | 1.96-fold decrease *(C. acnes)* | p=0.02 |
|  |  |  |  |  | Inverse Simpson | Increased 1.11-fold compared to baseline | * |  |  |
| Thompson, 2020 | 8 (minocycline) | RAGS (range) | 3 - 5 | - | Shannon | Increased compared to baseline and compared to acne-free controls | NS | Decrease *(C. acnes)* | p<0.05 |
| Chien, 2019 | 4 (minocycline) | No. of inflamed lesions (median) | 7 | 4.5 | Other | Decreased compared to baseline | NS | Decrease *(C. acnes)* | p<0.05 |
| Kelhälä, 2018 | 9 (lymecycline) | RAGS (mean) | 2.23 | 1.53 | Shannon / Inverse Simpson | Increased in cheek and back diversity compared to baseline Decreased in armpit diversity compared to baseline | * | -2.58 decrease | p=5.06e-3 |
|  | 13 (isotretinoin) | RAGS (mean) | 2.13 | 1.53 |  |  |  | -2.77 decrease | p=3.15e-6 |
| Dreno, 2016 | 26 (erythromycin) | GEA | 2.4 | - | Shannon index | - | - | -0.44% decrease | p<0.1 |
| McCoy, 2019 | 17 (isotretinoin) | GEA (mean) | 2.7 | 0.7 | Shannon | Increased compared to baseline | * | Lower in post-treatment | p<0.05 |
| Coughlin, 2017 | 5 (benzoyl peroxide) | CASS | 1.4 | 1 | Other^ǂ^ | Decreased compared to baseline | * | - |  |
|  | 3 (tretinoin) | CASS | 1.3 | 1 |  |  |  | - |  |
| Ahluwalia, 2019 | 51 (benzoyl peroxide) | IGA (mean) | 1.57 | - | Shannon | Decreased compared to baseline | NS | - |  |
| Karoglan, 2019 | 14 (benzoyl peroxide) | Leeds | 1.5 – 4 (range) | <3.1 (mean) | Shannon | Increased (2.6) compared to baseline (2.3) | - | - |  |

* p<0.05; ^ǂ^ based on number of observed species and phylogenetic diversity

*Abbreviations*: CASS, Comprehensive Acne Severity Scale; GEA, Global acne severity assessment; IGA, Investigator’s Global Severity; NS, Not Significant; RAGS, Leeds Revised Acne Grading System;

**Table S9**: PRISMA Checklist

| **Section/topic** | **#** | **Checklist item** | **Reported on page #** |
| --- | --- | --- | --- |
| **TITLE** | | |  |
| Title | 1 | Identify the report as a systematic review, meta-analysis, or both. | 1 |
| **ABSTRACT** | | |  |
| Structured summary | 2 | Provide a structured summary including, as applicable: background; objectives; data sources; study eligibility criteria, participants, and interventions; study appraisal and synthesis methods; results; limitations; conclusions and implications of key findings; systematic review registration number. | 2 |
| **INTRODUCTION** | | |  |
| Rationale | 3 | Describe the rationale for the review in the context of what is already known. | 4 |
| Objectives | 4 | Provide an explicit statement of questions being addressed with reference to participants, interventions, comparisons, outcomes, and study design (PICOS). | 4, 5 |
| **METHODS** | | |  |
| Protocol and registration | 5 | Indicate if a review protocol exists, if and where it can be accessed (e.g., Web address), and, if available, provide registration information including registration number. | 1, 4 |
| Eligibility criteria | 6 | Specify study characteristics (e.g., PICOS, length of follow-up) and report characteristics (e.g., years considered, language, publication status) used as criteria for eligibility, giving rationale. | 4, 5 |
| Information sources | 7 | Describe all information sources (e.g., databases with dates of coverage, contact with study authors to identify additional studies) in the search and date last searched. | 4 |
| Search | 8 | Present full electronic search strategy for at least one database, including any limits used, such that it could be repeated. | 5, Table S1-S3 |
| Study selection | 9 | State the process for selecting studies (i.e., screening, eligibility, included in systematic review, and, if applicable, included in the meta-analysis). | 5 |
| Data collection process | 10 | Describe method of data extraction from reports (e.g., piloted forms, independently, in duplicate) and any processes for obtaining and confirming data from investigators. | 5 |
| Data items | 11 | List and define all variables for which data were sought (e.g., PICOS, funding sources) and any assumptions and simplifications made. | 5 |
| Risk of bias in individual studies | 12 | Describe methods used for assessing risk of bias of individual studies (including specification of whether this was done at the study or outcome level), and how this information is to be used in any data synthesis. | 5 |
| Summary measures | 13 | State the principal summary measures (e.g., risk ratio, difference in means). | 5 |
| Synthesis of results | 14 | Describe the methods of handling data and combining results of studies, if done, including measures of consistency (e.g., I^2^) for each meta-analysis. | N/A |

Page 1 of 2

| **Section/topic** | **#** | **Checklist item** | **Reported on page #** |
| --- | --- | --- | --- |
| Risk of bias across studies | 15 | Specify any assessment of risk of bias that may affect the cumulative evidence (e.g., publication bias, selective reporting within studies). | N/A |
| Additional analyses | 16 | Describe methods of additional analyses (e.g., sensitivity or subgroup analyses, meta-regression), if done, indicating which were pre-specified. | N/A |
| **RESULTS** | | |  |
| Study selection | 17 | Give numbers of studies screened, assessed for eligibility, and included in the review, with reasons for exclusions at each stage, ideally with a flow diagram. | 6 |
| Study characteristics | 18 | For each study, present characteristics for which data were extracted (e.g., study size, PICOS, follow-up period) and provide the citations. | 6, Table 1, Table S4 |
| Risk of bias within studies | 19 | Present data on risk of bias of each study and, if available, any outcome level assessment (see item 12). | 6, Table S5 |
| Results of individual studies | 20 | For all outcomes considered (benefits or harms), present, for each study: (a) simple summary data for each intervention group (b) effect estimates and confidence intervals, ideally with a forest plot. | 6-8, Table 2 |
| Synthesis of results | 21 | Present results of each meta-analysis done, including confidence intervals and measures of consistency. | N/A |
| Risk of bias across studies | 22 | Present results of any assessment of risk of bias across studies (see Item 15). | N/A |
| Additional analysis | 23 | Give results of additional analyses, if done (e.g., sensitivity or subgroup analyses, meta-regression [see Item 16]). | N/A |
| **DISCUSSION** | | |  |
| Summary of evidence | 24 | Summarize the main findings including the strength of evidence for each main outcome; consider their relevance to key groups (e.g., healthcare providers, users, and policy makers). | 8-10 |
| Limitations | 25 | Discuss limitations at study and outcome level (e.g., risk of bias), and at review-level (e.g., incomplete retrieval of identified research, reporting bias). | 9 |
| Conclusions | 26 | Provide a general interpretation of the results in the context of other evidence, and implications for future research. | 8-10 |
| **FUNDING** | | |  |
| Funding | 27 | Describe sources of funding for the systematic review and other support (e.g., supply of data); role of funders for the systematic review. | 1 |

*From:*  Moher D, Liberati A, Tetzlaff J, Altman DG, The PRISMA Group (2009). Preferred Reporting Items for Systematic Reviews and Meta-Analyses: The PRISMA Statement. PLoS Med 6(7): e1000097. doi:10.1371/journal.pmed1000097

For more information, visit: **www.prisma-statement.org**.

Page 2 of 2
